# Genetic Networking of the *Bemisia tabaci* Cryptic Species Complex Reveals Pattern of Biological Invasions

**DOI:** 10.1371/journal.pone.0025579

**Published:** 2011-10-03

**Authors:** Paul De Barro, Muhammad Z. Ahmed

**Affiliations:** 1 CSIRO Ecosystem Sciences, Brisbane, Queensland, Australia; 2 Department of Genetics, University of Pretoria, Pretoria, South Africa; Michigan State University, United States of America

## Abstract

**Background:**

A challenge within the context of cryptic species is the delimitation of individual species within the complex. Statistical parsimony network analytics offers the opportunity to explore limits in situations where there are insufficient species-specific morphological characters to separate taxa. The results also enable us to explore the spread in taxa that have invaded globally.

**Methodology/Principal Findings:**

Using a 657 bp portion of mitochondrial cytochrome oxidase 1 from 352 unique haplotypes belonging to the *Bemisia tabaci* cryptic species complex, the analysis revealed 28 networks plus 7 unconnected individual haplotypes. Of the networks, 24 corresponded to the putative species identified using the rule set devised by Dinsdale et al. (2010). Only two species proposed in Dinsdale et al. (2010) departed substantially from the structure suggested by the analysis. The analysis of the two invasive members of the complex, Mediterranean (MED) and Middle East – Asia Minor 1 (MEAM1), showed that in both cases only a small number of haplotypes represent the majority that have spread beyond the home range; one MEAM1 and three MED haplotypes account for >80% of the GenBank records. Israel is a possible source of the globally invasive MEAM1 whereas MED has two possible sources. The first is the eastern Mediterranean which has invaded only the USA, primarily Florida and to a lesser extent California. The second are western Mediterranean haplotypes that have spread to the USA, Asia and South America. The structure for MED supports two home range distributions, a Sub-Saharan range and a Mediterranean range. The MEAM1 network supports the Middle East - Asia Minor region.

**Conclusion/Significance:**

The network analyses show a high level of congruence with the species identified in a previous phylogenetic analysis. The analysis of the two globally invasive members of the complex support the view that global invasion often involve very small portions of the available genetic diversity.

## Introduction

An enduring challenge within the context of cryptic species is the delimitation of individual species within the complex [1]–[3]. In this context, the absence of morphological characters with which to identify individual species has lead to the reliance on molecular tools to identify and delimit species [4]–[7]. In exploring the relationships between cryptic taxa, phylogenetic methods that assume bifurcating trees e.g. neighbor joining, maximum likelihood, maximum parsimony and minimum evolution are often used to explore these relationships however, gene evolution cannot always be represented in this manner. Rather, genealogies of closely related taxa may be multifurcated; descendant genes coexist with persistent ancestors producing reticulate relationships. To address this, networking approaches have been developed to estimate genealogies; these include pyramids technique [8], statistical geometry [9], split decomposition [10], the median networks [11], [12], median-joining networks approaches [13]–[14], molecular-variance parsimony [15], netting [16], likelihood network [17], reticulogram [18] and reticulate phylogeny [19]. More recently, Hart and Sunday (2007) [20] and Chen et al. (2010) [21] observed that the application of statistical parsimony network analytics to the question of species delimitation, identified a strong association between breaks in network connectivity and species level separation. Using mitochondrial cytochrome oxidase 1 (mtCOI) and the software TCS v1.21 [22] they calculated the maximum number of mutational steps needed to make a parsimonious connection between two haplotypes with the probability of 95%. These haplotypes were then linked using cladogram estimation analyses to form a network [23]. Where the number of steps needed to form a link exceeded the likelihood of 5%, those haplotypes remained unconnected. This method is particularly appropriate for population level analysis as it does not involve many of the assumptions of phylogenetic reconstruction methods e.g. it does not assume that the ancestral sequence is missing and does not require bifurcating relationships [24]. The use of statistical parsimony emphasizes what is shared among haplotypes that differ minimally rather than the differences among the haplotypes and provides an empirical assessment of deviations from parsimony. Using this approach Hart and Sunday (2007) [20] and Chen et al. (2010) [21] were able to predict species limits within species complexes that lacked sufficient species-specific morphological characters to separate individual taxa.

Since the early 1990s there has been the ongoing debate as to taxonomic identity of the whitefly, *Bemisia tabaci* (Gennadius) (Hemiptera: Aleyrodidae). In their recent review, De Barro et al. (2011) [25] explored the history of the debate as to whether *B. tabaci* was a complex species composed of biotypes or a complex composed of multiple species. They concluded that separation of different members of the *B. tabaci* complex using morphological or biological traits was not possible and that all reliable separations were being made on the basis of molecular data, primarily mtCOI. In particular, they noted that the Bayesian phylogenetic analyses of mtCOI haplotypes by Boykin et al. (2007) [26] and Dinsdale et al. (2010) [27] provided compelling evidence to support the case for a cryptic species complex. In particular, Dinsdale et al. (2010) [27] through the analysis of lineage divergence based on the Kimura-2 parameter (K2P) model of molecular evolution, that there was a distinct break in the pairwise frequency distribution at 3.5% sequence divergence and argued that this was evidence for species level separation. Goldstein & DeSalle (2011) [28] urge caution though in delimitation based on a single gene and argue that delimitation using single gene data is most compelling when used in concert with other data. The integration of other data was a key part of the review [25] which considered the evidence for species level separation in *B. tabaci*. Here, they observed that the separation identified in Dinsdale et al (2010) [27] was well supported by all the available mating compatibility data which showed that all crosses between putative species demonstrated either complete mating isolation or partial isolation resulting in reduced fitness in the progeny [29]–[34]. These studies suggested that *B. tabaci* was composed of at least 24 separate species while Hu et al. (2011) [35], using the approach outlined in Dinsdale et al. (2010) [27], increased the number to 28. The terminology used to describe *B. tabaci* species is that of Dinsdale et al. (2010) [27]. We refer to them as ‘putative species’ as this recognises the need for formal descriptions and additional corroborating evidence. The link between biotype terminology and the putative species terminology is detailed in Dinsdale et al (2010) [27] and De Barro et al. (2011) [25].

We have applied statistical parsimony network analysis using TCS to explore whether the approach supports the one used by Dinsdale et al. (2010) [27]. In addition, mtCOI has been widely used to explore the possible origins of invasive species [e.g. 36–38] and the analysis of network relationships provides a good opportunity to explore the geographic relationship of haplotypes and from this infer patterns of spread from their respective home ranges. In the context of the *B. tabaci* species complex, this is of considerable interest in regards to two members of the complex, Middle East-Asia Minor 1 (MEAM1, commonly known as the B biotype) and Mediterranean (MED, commonly known as the Q biotype) which have become global invaders through the trade in ornamentals 39,40.

## Results

### Mitochondrial cytochrome oxidase 1 haplotype network analysis

**Figure 1:**
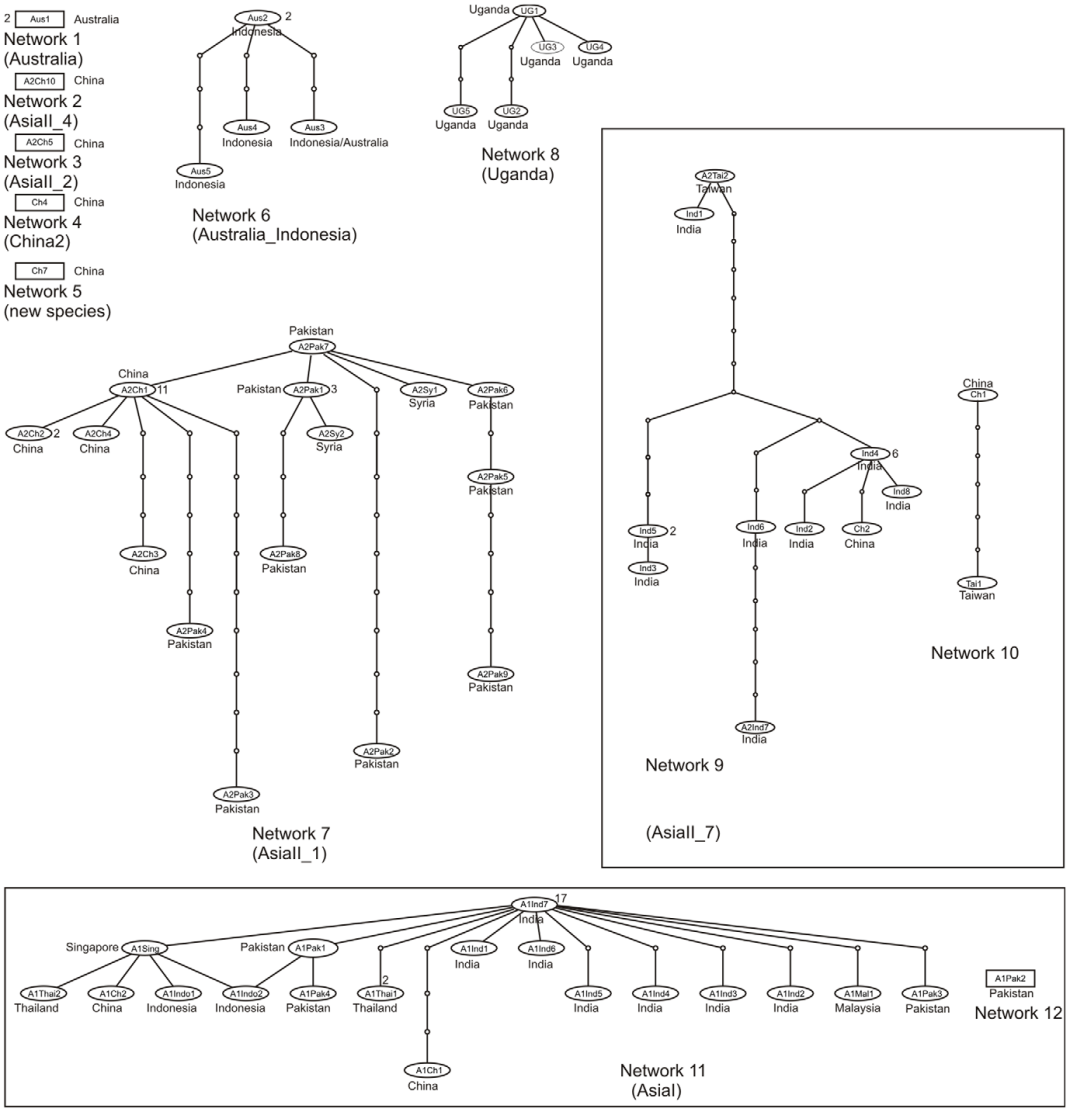
Networks 1–11 as per **Table 1**. Network analysis based on statistical parsimony [23] showing the genealogical relationships of the COI haplotypes in cryptic species of *Bemisia tabaci*. The Dinsdale putative species [27] are indicated in parentheses. Networks encompassed by a box are those Dinsdale putative species that the analysis suggests have additional species level separation. The names codes in the ovals refer to the individual haplotypes (see Table S1). The small circles indicate the presence of missing intermediates while the connections are based on the set of plausible solutions with a 95% of parsimony probability. The number of sequences for each haplotype where n>1 is indicated next to the node; nodes without a number are n = 1.

**Figure 2:**
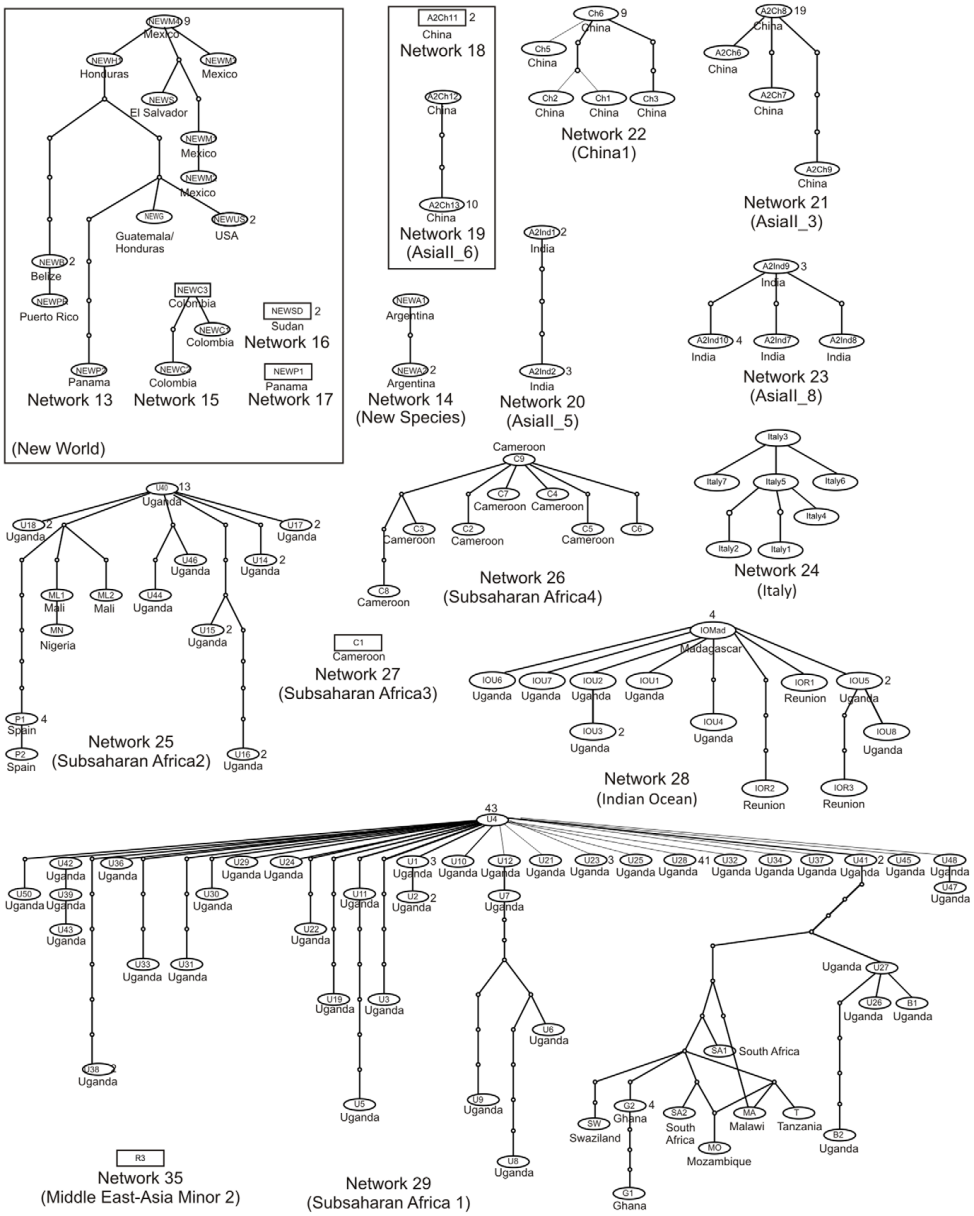
Networks 12–29 and 35 as per **Table 1**. Network analysis based on statistical parsimony [23] showing the genealogical relationships of the COI haplotypes in cryptic species of *Bemisia tabaci*. The Dinsdale putative species [27] are indicated in parentheses. Networks encompassed by a box are those Dinsdale putative species that the analysis suggests have additional species level separation. The names codes in the ovals refer to the individual haplotypes (see Table S1). The small circles indicate the presence of missing intermediates while the connections are based on the set of plausible solutions with a 95% of parsimony probability. The number of sequences for each haplotype where n>1 is indicated next to the node; nodes without a number are n = 1. Networks 31–24 which related to the Mediterranean (networks 30, 31) and Middle East - Asia Minor 1 (networks 32–34) putative species are shown in Figs 3 and 4, respectively.

**Figure 3:**
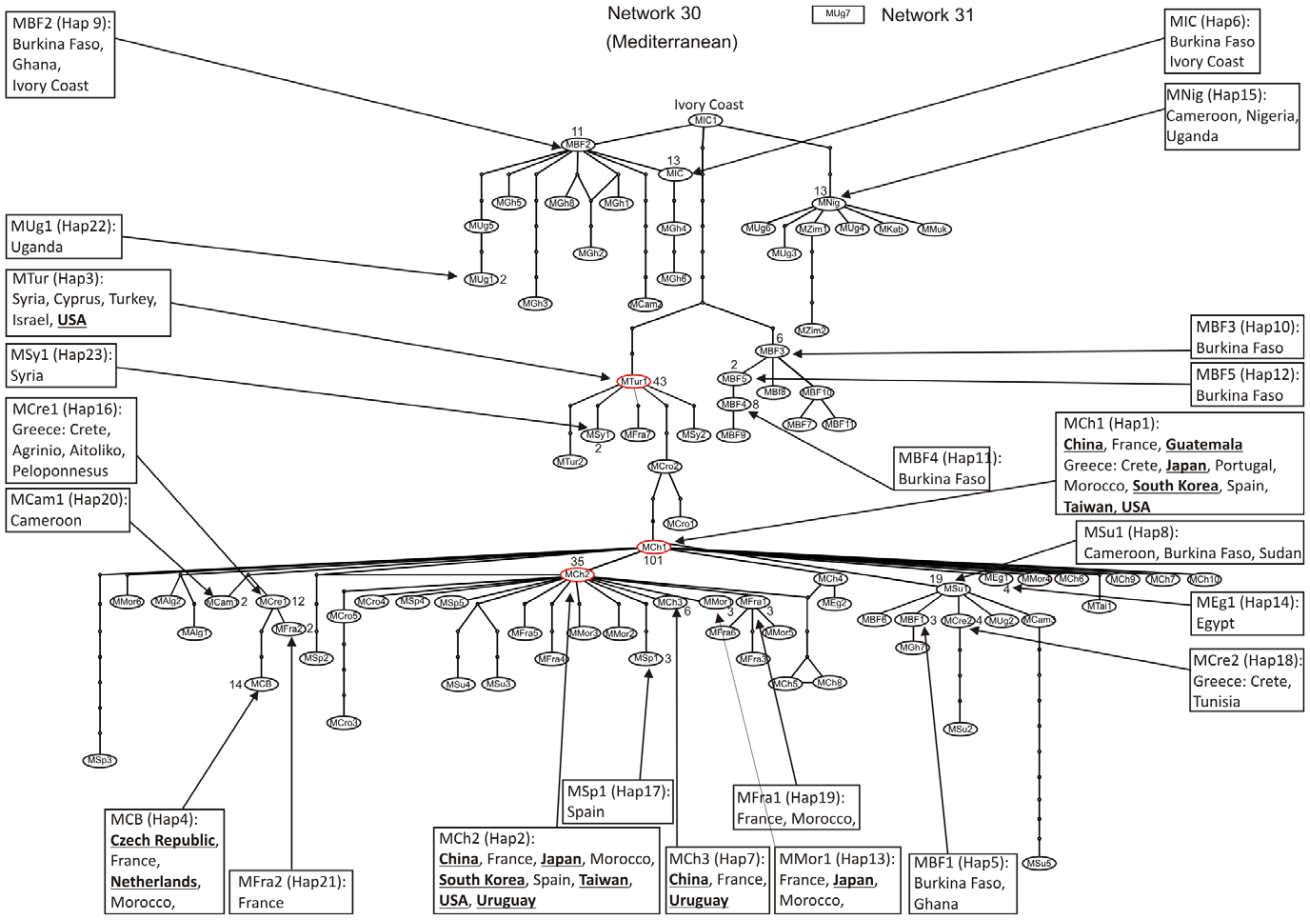
Network analysis based on statistical parsimony [**23**] showing the genealogical relationships of the COI haplotypes representing the Mediterranean putative species. The two networks are numbered 30 and 31. Network 30 represents 85 of the 86 haplotypes assigned to MED using the approach outlined in [27]. The names codes in the ovals refer to the individual haplotypes (see Table S1). The small circles indicate the presence of missing intermediates while the connections are based on the set of plausible solutions with a 95% of parsimony probability. Each of the haplotypes (23 in total, see Table 4) for which there were at least two identical representatives in GenBank are indicated by an arrow linked to a box which details the countries where the haplotype was found; countries that are in bold and underlined are those which indicate an invasion. The ovals coloured in red indicate those haplotypes that together represent >80% of the GenBank records in the invaded ranges. The number of sequences for each haplotype where n>1 is indicated next to the node; nodes without a number are n = 1.

**Figure 4:**
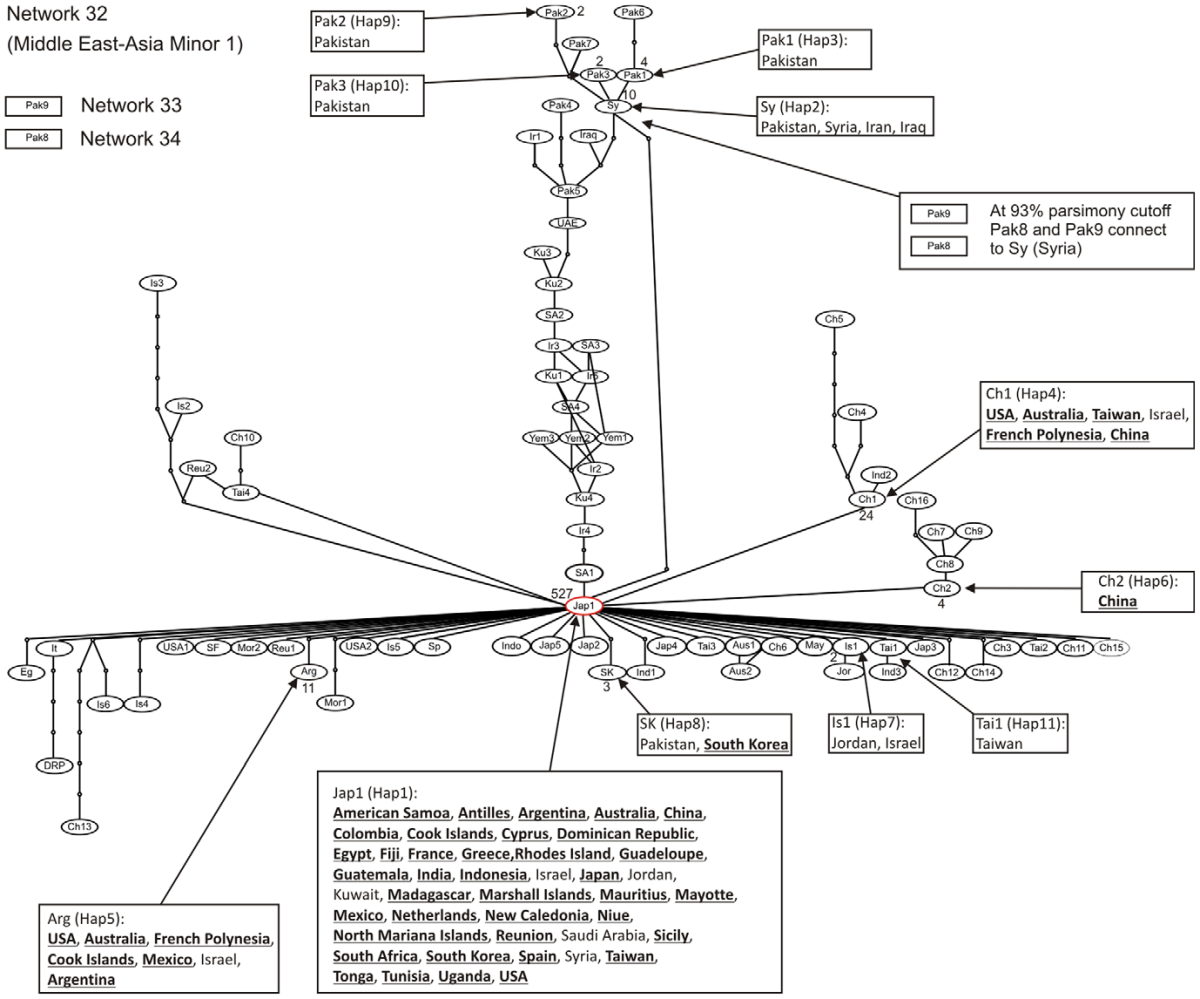
Network analysis based on statistical parsimony [**23**] showing the relationships of the mtCOI haplotypes representing the Middle East – Asia Minor 1 putative species. The three networks are numbered 32, 33 and 34. Network 32 represents 79 of the 80 haplotypes assigned to MEAM1 using the approach outlined in Dinsdale et al. (2010) [27]. The name codes in the ovals refer to the individual haplotypes (see Table S1). The small circles indicate the presence of missing intermediates while the connections are based on the set of plausible solutions with a 95% of parsimony probability. Each of the haplotypes (11 in total, see Table 5) for which there were at least two identical representatives in GenBank are indicated by an arrow linked to a box which details the countries where the haplotype was found; countries that are in bold and underlined are those which indicate an invasion. The oval coloured in red indicates the haplotype that represents >80% of the GenBank records in the invaded ranges. The number of sequences for each haplotype where n>1 is indicated next to the node; nodes without a number are n = 1.

Statistical parsimony analysis of the 352 unique haplotypes revealed the presence of 35 distinct networks (Figs 1, 2). Of the 352 haplotypes all, but three, were able to be assigned to one of the 24 putative species. The three remaining haplotypes, EU192051 (China) and, AF340212 and AF340213 (Argentina), diverged by >3.5% and so were considered to be new putative species based on the rule set in Dinsdale et al. (2010) [27]. There was a high degree of agreement between the species level delimitation based on the network analysis and that identified by the 3.5% break in sequence divergence in Dinsdale et al. (2010) [27]. Of the 24 putative species proposed by Dinsdale et al. (2010) [27], 18 corresponded directly to a single network (Table 1, Figs 1, 2). Of the remaining six putative species three, AsiaI (Fig. 1), AsiaII_6 (Fig. 2) and MED (Fig. 3) each consisted of a network plus one unconnected haplotype and MEAM1 (Fig. 4) split into one network plus two unconnected haplotypes (Table 1). For AsiaI the unconnected haplotype was from Pakistan (A1Pak2, AJ510066), for AsiaII_6 from China (A2Ch11, AJ784261), for MED from Uganda (MUg7, AY903565) and for Middle East-Asia Minor 1 the two unconnected haplotypes were both from Pakistan (Pak8, GU977267; Pak9 GU977268). Of the two remaining putative Dinsdale species, AsiaII_7 (Fig. 1) split into two separate networks and New World (Fig. 2) split into two networks, plus two unconnected haplotypes (Table 1). In the case of AsiaII_7, one network contained haplotypes from India and China while the other had haplotypes from China only (Fig. 1). For the New World, one network contained haplotypes from Belize, El Salvador, Guatemala, Honduras, Mexico, Puerto Rico and the south western USA while the second had haplotypes from Colombia; haplotypes from Panama (NewP1, DQ130060) and Sudan (NewSD, EU760727) were unconnected (Fig. 2).

**Table 1:** The relationship between the Dinsdale putative species [27] and the number of networks identified using statistical parsimony; the number haplotypes within each network and mean Kimura-2-parameter (K2P) distances for mtCOI within each network where n>1.

Dinsdale species based on 3.5% sequence divergence	Number of networks	Network identification number	Haplotypes per network	K2P distances range	K2P distances mean
AsiaI	2	11	18	0.002–0.015	0.006
		12	1	-	-
AsiaII_1	1	7	15	0.002–0.026	0.011
AsiaII_2	1	3	1	-	-
AsiaII_3	1	21	4	0.002–0.009	0.005
AsiaII_4	1	2	1	-	-
AsiaII_5	1	20	2	0.006	0.006
AsiaII_6	2	18	2	0.031	0.005
		19	1	-	-
AsiaII_7	2	9	10	0.002–0.026	0.013
		10	2	0.009	0.009
AsiaII_8	1	23	4	0.003–0.006	0.005
Australia	1	1	1	-	-
Australia/Indonesia	1	6	4	0.009–0.011	0.008
China 1	1	22	5	0.002–0.009	0.006
China 2	1	4	1	-	-
New species China	1	5	1	-	-
Indian Ocean	1	28	12	0.002–0.012	0.005
Italy	1	24	7	0.002–0.006	0.004
Mediterranean	2	30	85	0.002–0.042	0.018
		31	1	-	-
Middle East–Asia Minor 1	3	32	78	0.002–0.033	0.009
		33	1	-	-
		34	1	-	-
Middle East–Asia Minor 2	1	35	1	-	-
New World	4	13	11	0.002–0.017	0.012
		15	3	0.002–0.017	0.005
		16	1	-	-
		17	1	-	-
New species Argentina	1	14	2	0.003	0.003
SubSaharan Africa 1	1	29	48	0.002–0.031	0.012
SubSaharan Africa 2	1	25	13	0.002–0.023	0.01
SubSaharan Africa 3	1	27	1	-	-
SubSaharan Africa 4	1	26	8	0.002–0.009	0.005
Uganda	1	8	5	0.002–0.006	0.005
Overall Average					0.137

The identification number for the network which relates to that in Figs 1, 2, 3, 4 is also indicated.

Table 2 provides the range of Kimura's 2-parameter (K2P) divergences for each unconnected haplotype or network that was unconnected to the network representing each of the six Dinsdale putative species. In each case the minimum K2P divergence ranged from 1.9% to 3.3% while the maximum ranged from 3.0% to 6.6% and averaged 2.5% to 4.7%. The mean genetic distances (K2P) within putative species for each of networks that represented the different Dinsdale putative species ranged from 0.3% to 1.8% (Table 1) while distances between networks were >2.0% with a maximum of 24.4% between network 8 and network 15 (Table 3).

**Table 2:** The six Dinsdale putative species [27] which the TCS network analysis showed were composed of two of more networks.

Dinsdale species based on 3.5% sequence divergence	Network code	Accession numbers	K2P range	Mean K2P divergence from main network
AsiaI	A1Pak2	AJ510066	2.0%–3.0%	2.5%
AsiaII_7	Tai1	AY686075	1.9%–3.9%	2.5%
	Ch1	AY686064		
AsiaII_6	A2Ch11	AJ784261	3.0%–3.1%	3.0%
Mediterranean	MUg7	AY903565	2.5%–6.6%	4.7%
Middle East-Asia Minor 1	Pak8	GU977267	2.0%–3.9%	2.9%
	Pak9	GU977268	2.3%–4.0%	3.1%
New World	NewSD	EU760727	3.3%–4.6%	3.8%
New World	NewC1	AJ550167	2.1%–3.6%	3.0%
	NewC2	AJ550168		
	NewC3	EU427728		
New World	NewP1	DQ130060	2.2%–3.0%	2.4%

The network codes and accession numbers for the haplotypes that were found not to be connected to the main network are shown as well as the Kimura-2-parameter (K2P) divergence range and mean K2P divergence from the main network.

**Table 3:** Between network Kimura-2-parameter genetic distances.

	N11	N12	N7	N3	N21	N2	N20	N18	N19	N9	N10	N23	N6	N1	N22	N4	N5	N28
N11	-																	
N12	2.5	-																
N7	16.0	17.0	-															
N3	14.2	15.2	6.1	-														
N21	17.6	18.4	13.9	7.3	-													
N2	16.6	17.8	12.1	5.4	4.3	-												
N20	17.1	18.3	10.4	9.2	13.1	11.9	-											
N18	17.3	17.9	12.2	11.8	14.5	13.9	6.9	-										
N19	16.5	18.1	12.2	12.0	15.1	14.2	6.9	3.0	-									
N9	14.1	14.5	10.3	10.5	12.9	12.2	11.6	11.2	11.3	-								
N10	14.5	15.2	9.0	9.0	11.6	10.8	10.5	10.5	10.7	2.5	-							
N23	15.3	16.6	13.8	11.3	11.8	13.4	12.6	13.9	13.4	12.1	11.2	-						
N6	14.6	16.2	17.9	14.9	17.6	17.4	16.8	17.7	17.8	18.4	16.9	17.7	-					
N1	15.0	16.7	18.5	15.2	18.0	18.0	17.5	18.0	18.1	18.7	17.1	18.0	4.0	-				
N22	13.4	14.9	15.7	12.8	15.4	14.5	13.8	14.3	14.6	14.7	12.9	15.0	14.9	14.5	-			
N4	13.0	14.3	15.7	12.8	15.1	14.0	13.8	14.8	15.3	13.6	11.9	14.7	14.7	14.3	4.6	-		
N5	14.0	15.3	15.6	12.2	15.1	15.7	15.2	15.3	15.1	13.8	12.8	12.5	12.8	11.8	11.5	10.8	-	
N28	15.1	16.1	17.2	14.0	17.4	16.9	17.9	18.4	18.9	17.4	15.7	16.9	18.5	18.2	16.7	15.8	18.1	-
N30	16.7	17.2	17.9	13.5	17.9	16.9	18.4	18.3	17.3	17.2	15.7	16.6	18.6	18.0	17.0	16.5	16.2	7.1
N31	18.8	19.4	20.1	15.8	20.7	19.1	20.1	21.1	20.1	18.6	17.6	18.6	20.3	19.8	18.5	18.1	17.0	9.7
N24	14.3	15.5	14.5	12.2	12.7	13.4	13.8	13.4	13.8	13.3	11.4	12.4	13.2	12.7	12.2	11.4	13.6	15.4
N32	16.5	17.1	17.7	11.8	17.8	15.4	17.5	18.6	17.3	17.9	16.1	16.2	17.9	18.0	16.6	15.2	15.4	8.1
N33	17.7	18.3	19.5	13.9	19.8	17.3	19.4	20.5	19.5	19.4	17.7	18.5	19.7	19.8	18.7	17.9	17.3	9.8
N34	16.5	17.1	18.1	12.3	18.3	15.9	17.5	18.6	17.7	18.3	16.7	16.7	17.7	17.8	16.9	15.6	15.6	8.2
N35	15.7	16.4	16.2	12.3	16.7	15.7	16.9	16.6	16.1	16.4	15.1	14.9	17.9	17.6	15.9	15.6	15.0	7.0
N1	16.0	17.1	19.4	16.8	18.0	18.7	18.4	19.7	20.6	18.5	18.2	18.3	19.3	18.9	16.6	16.0	17.8	19.1
N13	16.1	17.3	19.2	16.6	18.2	18.1	18.2	19.5	20.4	18.9	18.4	18.4	20.0	19.8	17.3	16.9	18.9	18.4
N15	16.8	16.8	20.7	18.4	19.8	19.5	19.6	21.4	21.4	19.8	19.2	20.6	22.6	22.9	19.4	18.1	20.9	18.8
N16	18.0	18.9	21.3	18.4	20.1	20.2	20.1	20.8	21.7	21.1	20.5	20.4	21.2	20.7	19.2	18.8	20.3	20.5
N17	16.5	17.5	19.7	16.9	17.2	18.0	17.5	18.4	19.0	18.1	17.8	17.7	20.7	20.6	17.4	16.0	18.6	18.5
N29	18.2	20.2	21.3	17.8	18.4	19.0	20.9	20.8	21.2	21.3	20.1	19.0	19.3	19.4	20.0	19.3	19.1	18.9
N25	19.7	22.0	21.1	18.1	19.3	19.5	21.2	20.5	20.8	20.6	20.2	19.4	19.9	19.7	19.3	19.7	18.0	20.2
N27	16.4	18.1	19.4	16.8	18.3	18.4	20.4	20.4	21.1	19.9	19.2	17.2	18.8	18.5	18.6	18.0	18.0	18.6
N26	17.9	19.6	20.5	17.5	19.2	18.9	20.2	21.0	21.6	19.8	19.0	19.1	18.7	18.6	19.9	19.7	18.3	18.8
N8	22.4	24.2	21.8	19.7	23.3	22.4	21.2	22.9	22.9	23.0	22.6	21.0	22.0	21.3	20.5	20.1	19.7	21.8

	N30	N31	N24	N32	N33	N34	N35	N1	N13	N15	N16	N17	N29	N25	N27	N26	N8	
N11																		
N12																		
N7																		
N3																		
N21																		
N2																		
N20																		
N18																		
N19																		
N9																		
N10																		
N23																		
N6																		
N1																		
N22																		
N4																		
N5																		
N28																		
N30	-																	
N31	4.7	-																
N24	15.8	17.6	-															
N32	5.6	7.8	15.4	-														
N33	7.4	9.8	17.7	2.9	-													
N34	5.7	7.9	16.0	1.1	2.0	-												
N35	4.1	6.7	14.8	4.4	6.6	4.8	-											
N1	17.4	19.2	14.8	18.9	17.2	17.3	16.7	-										
N13	17.3	20.0	15.7	18.7	16.7	17.7	17.3	2.9	-									
N15	18.5	20.9	17.0	19.7	18.0	18.4	18.0	6.2	2.5	-								
N16	19.2	21.9	17.6	20.7	19.0	19.5	19.0	3.9	2.4	7.3	-							
N17	18.0	19.6	14.1	18.9	16.8	17.6	16.8	4.1	3.8	5.6	5.4	-						
N29	18.9	21.5	15.3	19.8	17.4	18.4	17.4	16.8	16.6	20.3	18.4	17.4	-					
N25	18.9	21.0	16.6	19.6	17.5	18.3	17.5	17.4	17.3	20.7	19.2	17.8	8.9	-				
N27	18.7	21.7	15.9	19.5	17.6	18.1	17.6	16.0	15.8	19.2	17.6	17.4	6.6	8.0	-			
N26	18.6	20.4	15.2	20.1	17.6	18.7	17.6	16.3	16.3	20.1	18.3	17.1	8.0	9.2	7.4	-		
N8	20.7	22.1	21.0	22.3	19.7	20.8	19.7	21.3	20.2	24.4	21.9	21.6	20.7	20.7	20.5	21.4	-	

N1-N35 indicates network identity, see Table 2 to relate network numbers to Dinsdale putative species [27]; network numbering follows the order of Table 2.

### Haplotype diversity and distribution for globally invasive *B. tabaci*

Of the 660 MEAM1 sequences, 601 (0.911) came from the invaded range and 59 (0.089) from the home range. Furthermore, of the 80 unique haplotypes, 40 came from the home range, 44 from the invaded range, four from both, 36 from the home range only and 40 from the invaded range only. Of the 374 MED sequences, 153 (0.409) came from the invaded range and 221 (0.591) from the home range. Of the 86 unique haplotypes, 76 came from the home range, 12 from the invaded range, four from both, 72 from the home range only and eight from the invaded range only.

For MED, the search of GenBank identified 86 unique haplotypes. Of these 23 (MED-Hap1 to MED-Hap23) were represented by at least two sequences while the remaining 63 were represented by a single sequence (Table S1, Table 4, Fig. 3) and 1, MUg7 (Uganda) was shown not to be connected to the network. The network analysis suggested a complex structure for MED. There are two distinct elements to the network. The first element is composed primarily of countries from Sub-Saharan Africa (Fig. 3). This network consists of a hub (Ivory Coast, MIC2) with one branch consisting of primarily West African countries (Burkina Faso, Cameroon, Ghana, Ivory Coast) as well as Uganda and a branch consisting of both East and West African countries (Cameroon, Nigeria, Uganda, Zimbabwe). It is interesting to note that the primarily West African branch contains haplotypes that have retained the capacity to induce silverleafing which has been lost throughout the rest of the network (Fig. 3). A third branch which splits into a branch containing haplotypes from Burkina Faso and a branch with haplotypes from Croatia, Cyprus, Egypt, France, Israel, Syria, Turkey and USA (Fig 3). This Mediterranean branch acts as the conduit to the second element of the network. The network structure suggests a connection from Sub-Saharan Africa through Turkey/Syria/Israel (MTur) to the eastern Mediterranean (Fig. 3). The primarily eastern Mediterranean portion of the network then connects through Croatia to the node MCh1. This haplotype occurs in the western Mediterranean countries of France, Morocco, Portugal and Spain as well to the eastern Mediterranean island of Crete and countries well outside the region, China, Guatemala, Japan, South Korea, Taiwan and USA (Fig. 3). MCh1, forms a hub that connects an array of closely connected haplotypes. These haplotypes mostly occur either in the Mediterranean (Algeria, Crete, Croatia, Egypt, France, Morocco, Spain, Syria, Tunisia) or occur in countries well outside the region (China, Czech Republic, Japan, Netherlands, South Korea, Taiwan, Uruguay, USA) (Table 4, Fig. 3). The exceptions to these are haplotypes that come from Cameroon, Sudan and a cluster of connected haplotypes stemming from MSu1 which include the countries of (Burkina Faso, Cameroon, Ghana, Greece, Sudan, Tunisia, Uganda) (Table 4, Fig. 3). The network structure supports the idea that MED has a home range that extends from Sub-Saharan Africa through into the Mediterranean region.

**Table 4:** The number of haplotypes in GenBank that were assigned to the Mediterranean putative species.

Haplotype	Number of sequences in GenBank	Percentage of total	Countries in presumed home range	Countries in invaded range
MED-1	101	27.0	France, Greece: Crete, Morocco, Portugal, Spain	China, Guatemala, South Korea, Taiwan, USA, Japan
MED-2	35	9.4	France, Morocco, Spain	China, Japan, South Korea, Taiwan, Uruguay, USA
MED-3	43	11.5	Cyprus, Israel, Syria, Turkey	USA
MED-4	14	3.7	France, Morocco	Czech Republic, Netherlands
MED-5	3	0.8	Burkina Faso, Ghana	
MED-6	13	3.5	Burkina Faso, Ivory Coast	
MED-7	6	1.6	France	China, Uganda
MED-8	19	5.1	Burkina Faso, Cameroon, Sudan	
MED-9	11	2.9	Burkina Faso, Ghana, Ivory Coast	
MED-10	6	1.6	Burkina Faso	
MED-11	8	2.1	Burkina Faso	
MED-12	2	0.5	Burkina Faso	
MED-13	3	0.8	France, Morocco	Japan
MED-14	4	1.1	Egypt	
MED-15	13	3.5	Cameroon, Nigeria, Uganda	
MED-16	12	3.2	Greece	
MED-17	3	0.8	Spain	
MED-18	4	1.1	Greece, Tunisia	
MED-19	3	0.8	France, Morocco	
MED-20	2	0.5	Cameroon	
MED-21	2	0.5	France	
MED-22	2	0.5	Uganda	
MED-23	2	0.5	Syria	
singleton	63	16.8		
Total	374			

There was a total of 86 unique haplotypes. The number of identical haplotype accessions is indicated for each of the unique haplotypes as well as the number of haplotypes for which there was only one accession (singleton). The source country for each accession is indicated along with whether or not it occurred in the presumed home range.

The global pattern of spread beyond this assumed home range is more complex than for MEAM1. Of the 85 haplotypes considered, 14 have spread outside this region. In the case of the USA, three haplotypes have been recorded. MSy (MED-Hap 3, Table 4, Fig. 3) which also occurs in Cyprus, Israel, Syria and Turkey suggests an introduction from the eastern Mediterranean into the USA. However, the presence in the USA of MCh1 (Table 4, Fig. 3) which is associated to the assumed home range through France, Greece: Crete, Morocco, Portugal, Spain, suggests a second invasion from the western Mediterranean has also occurred. This haplotype also now occurs in China, Guatemala, Japan, South Korea and Taiwan. MCh2 (MED-Hap 2, Table 4, Fig. 3) which is also associated with the western Mediterranean (France, Morocco, Spain) and now also occurs in the USA further supports the idea that the USA has experienced two waves of invasion. The haplotype also occurs in China, Japan, South Korea, Taiwan and Uruguay. In the case of mainland China, the same three haplotypes that have invaded the USA also occur there along with one haplotype (MFra4) which also occurs in France as well as Uruguay suggesting both eastern and western Mediterranean origin invasions into China (Table 4, Fig. 3). A further seven haplotypes (MCh4, MCh5, MCh6, MCh7, MCh8, MCh9, MCh10) occur in China that so far have been found nowhere else (Table 4, Fig. 3). The remaining haplotypes that occur outside the assumed home range are MCBH4 (MED-Hap 4, Table 4) Czech Republic, France, Morocco and the Netherlands; MMor1 (Table 4, Fig 3) France, Morocco and Japan both of which again suggest western Mediterranean origins and two haplotypes found only in Japan and Taiwan. What is of particular interest in regards to the patterns of global invasion is that with the exception of the eastern Mediterranean, MTur, all haplotypes that have been found outside the Mediterranean/Sub-Saharan Africa regions were from the western Mediterranean and belonged either to the haplotype MCh1 or were very closely related to this haplotype (Table 4, Fig. 3). This mirrors the pattern observed for MEAM1 in that a single haplotype has been hub from which all invading haplotypes stem (Figs 3, 4).

In the case of MEAM1, the search of GenBank identified 80 unique haplotypes, two of which were subsequently shown not to form part of the MEAM1 network. Of the remaining 80 haplotypes, 11 (MEAM1-Hap 1 to MEAM1-Hap 11) were represented by at least two sequences while the remaining 69 were represented by a single sequence (Table S1, Table 5, Fig. 4). The network analysis shows one branch composed of haplotypes from either the Middle East (Kuwait, Saudi Arabia, Syria, United Arab Emirates, Yemen) or Asia Minor (Iran, Iraq, Pakistan); none of the haplotypes occur outside the assumed home range of MEAM1 (Table 5, Fig. 4). This branch connects to the haplotype referred to as Jap1 (Table 5, Fig. 4). This haplotypes has spread widely beyond the assumed home range of MEAM1 to 37 countries across Africa, Europe, Asia, Oceania and the New World and accounts 79.8% of the mtCOI sequences in GenBank. Jap1 forms a central hub from which all the remaining 51 haplotypes radiate (Fig. 4). Of these, 11/51 come from either Israel (8), Jordan (2) or Pakistan (1) while a further 44/51 haplotypes come from countries beyond the assumed home range (Table 5, Fig. 4). Of these, three, Arg (MEAM1-Hap5, Argentina, Australia, Cook Islands, French Polynesia, Mexico, Israel and USA, Table 5, Fig. 4), SK (MEAM1-Hap 8, Pakistan and South Korea, Table 5, Fig. 4) and Ch1 (MEAM1-Hap 4, Australia, China, French Polynesia, Israel, Taiwan and USA) have direct links to the assumed home range through Israel and together account for a further 5.6% of the sequences in GenBank. This indicates that from a global invasion perspective, four haplotypes account for approximately 85% of all invasion records. What is particularly interesting is that Israel does not feature in any of the countries in the branch composed of haplotypes only from the home range; Israel first appears in Jap1. Another feature of the network is that of the 78 sequences, 27 are directly connected to Jap1 with a further 12 separated by a single node.

**Table 5:** The number of haplotypes in GenBank that were assigned to the Middle East - Asia Minor 1 putative species.

Haplotype	Number of sequences in GenBank	Percentage of total	Countries in presumed home range	Countries in invaded range
MEAM1- 1	527	79.7	Israel, Jordan, Kuwait, Saudi Arabia, Syria	American Samoa, Antilles, Argentina, Australia, China, Colombia, Cook Islands, Cyprus, Dominican Republic, Egypt, Fiji, France, Greece: Rhodes Island, Guadeloupe, Guatemala, India, Indonesia, Japan, Madagascar, Marshall Islands, Mauritius, Mayotte, Mexico, Netherlands, New Caledonia, Niue, North Mariana Islands, Reunion, Sicily, South Africa, South Korea, Spain, Taiwan, Tonga, Tunisia, Uganda, USA, Cyprus; Dominican Republic
MEAM1- 2	10	1.5	Syria, Iran, Iraq, Pakistan	
MEAM1- 3	4	0.6	Pakistan	
MEAM1- 4	24	3.7	Israel	Australia, China, French Polynesia, Taiwan, USA
MEAM1- 5	11	1.7	Israel	Argentina, Cook Islands, French Polynesia, Japan, Mexico, USA
MEAM1- 6	4	0.6		China
MEAM1- 7	2	0.3	Israel, Jordon	
MEAM1- 8	3	0.3	Syria	South Korea
MEAM1- 9	2	0.3	Pakistan	
MEAM1- 10	2	0.3	Pakistan	
MEAM1- 11	2	0.3		Taiwan
singleton	69	11.5		
Total	660			

There was a total of 80 unique haplotypes. The number of identical haplotype accessions is indicated for each of the unique haplotypes as well as the number of haplotypes for which there was only one accession (singleton). The source country for each accession is indicated along with whether or not it occurred in the presumed home range.

The results Φst analysis and AMOVA support there being a complete genetic break among the 35 independent networks (Table 6) and so provides additional support for existence of distinct cryptic species. The AMOVA showed a very high percentage of variation among networks, 92.01% whereas the within network variation is quite low, 7.99%.

**Table 6:** *Bemisia tabaci* pairwise Φst values among 35 independent networks shown in Figs 1, 2.

	N11	N12	N7	N3	N21	N2	N20	N18	N19	N9	N10	N23	N1	N6	N22	N4	N5	N28
N11	0.00																	
N12	0.77	0.00																
N7	0.94	0.93	0.00															
N3	0.95	1.00	0.81	0.00														
N21	0.96	0.97	0.92	0.92	0.00													
N2	0.96	1.00	0.90	1.00	0.87	0.00												
N20	0.96	0.96	0.89	0.93	0.95	0.94	0.00											
N18	0.96	0.97	0.91	0.96	0.96	0.96	0.92	0.00										
N19	0.96	1.00	0.90	1.00	0.96	1.00	0.90	0.85	0.00									
N9	0.94	0.90	0.88	0.87	0.91	0.89	0.89	0.89	0.88	0.00								
N10	0.95	0.93	0.87	0.89	0.94	0.91	0.92	0.93	0.90	0.51	0.00							
N23	0.96	0.97	0.92	0.96	0.95	0.96	0.96	0.96	0.96	0.90	0.94	0.00						
N1	0.95	1.00	0.92	1.00	0.96	1.00	0.95	0.97	1.00	0.91	0.93	0.97	0.00					
N6	0.96	0.95	0.94	0.95	0.96	0.95	0.95	0.96	0.95	0.93	0.95	0.96	0.94	0.00				
N22	0.95	0.96	0.93	0.95	0.96	0.96	0.95	0.96	0.96	0.92	0.95	0.96	0.96	0.95	0.00			
N4	0.95	1.00	0.92	1.00	0.96	1.00	0.95	0.97	1.00	0.90	0.92	0.97	1.00	0.94	0.88	0.00		
N5	0.95	1.00	0.92	1.00	0.96	1.00	0.95	0.97	1.00	0.90	0.92	0.96	1.00	0.93	0.95	1.00	0.00	
N28	0.96	0.97	0.95	0.96	0.97	0.97	0.97	0.97	0.97	0.95	0.96	0.97	0.97	0.97	0.97	0.97	0.97	0.00
N30	0.90	0.88	0.90	0.86	0.89	0.88	0.89	0.89	0.88	0.89	0.88	0.89	0.89	0.90	0.89	0.88	0.88	0.77
N31	0.97	1.00	0.94	1.00	0.97	1.00	0.96	0.97	1.00	0.92	0.94	0.97	1.00	0.96	0.97	1.00	1.00	0.95
N24	0.96	0.97	0.93	0.96	0.96	0.97	0.97	0.97	0.97	0.92	0.95	0.96	0.97	0.96	0.96	0.96	0.97	0.97
N32	0.95	0.94	0.94	0.92	0.95	0.94	0.95	0.95	0.95	0.94	0.94	0.94	0.94	0.95	0.94	0.94	0.94	0.90
N33	0.96	1.00	0.94	1.00	0.97	1.00	0.96	0.97	1.00	0.93	0.94	0.97	1.00	0.96	0.97	1.00	1.00	0.95
N34	0.96	1.00	0.94	1.00	0.97	1.00	0.96	0.97	1.00	0.92	0.94	0.97	1.00	0.96	0.97	1.00	1.00	0.95
N35	0.96	1.00	0.92	1.00	0.96	1.00	0.96	0.97	1.00	0.91	0.93	0.97	1.00	0.95	0.96	1.00	1.00	0.93
N13	0.95	0.94	0.94	0.94	0.95	0.94	0.94	0.95	0.94	0.93	0.94	0.95	0.94	0.95	0.95	0.94	0.94	0.96
N15	0.96	0.98	0.94	0.98	0.97	0.98	0.97	0.98	0.98	0.93	0.97	0.98	0.98	0.97	0.97	0.98	0.98	0.97
N16	0.96	1.00	0.94	1.00	0.97	1.00	0.96	0.98	1.00	0.93	0.95	0.97	1.00	0.96	0.97	1.00	1.00	0.97
N17	0.96	1.00	0.94	1.00	0.96	1.00	0.96	0.97	1.00	0.92	0.94	0.97	1.00	0.96	0.96	1.00	1.00	0.97
N14	0.96	0.98	0.94	0.98	0.97	0.98	0.97	0.98	0.98	0.93	0.96	0.98	0.98	0.97	0.97	0.98	0.98	0.97
N25	0.95	0.94	0.94	0.94	0.94	0.94	0.95	0.95	0.94	0.94	0.94	0.95	0.94	0.95	0.95	0.94	0.94	0.95
N29	0.94	0.94	0.94	0.93	0.93	0.93	0.94	0.94	0.93	0.93	0.93	0.93	0.93	0.94	0.93	0.93	0.93	0.94
N27	0.96	1.00	0.94	1.00	0.97	1.00	0.97	0.97	1.00	0.93	0.95	0.97	1.00	0.95	0.97	1.00	1.00	0.97
N26	0.96	0.97	0.95	0.97	0.97	0.97	0.97	0.97	0.97	0.95	0.97	0.97	0.97	0.96	0.97	0.97	0.97	0.97
N8	0.97	0.98	0.95	0.97	0.97	0.97	0.97	0.98	0.97	0.95	0.97	0.97	0.98	0.97	0.97	0.97	0.97	0.97

	N30	N31	N24	N32	N33	N34	N35	N13	N15	N16	N17	N14	N25	N29	N27	N26	N8	
N11																		
N12																		
N7																		
N3																		
N21																		
N2																		
N20																		
N18																		
N19																		
N9																		
N10																		
N23																		
N1																		
N6																		
N22																		
N4																		
N5																		
N28																		
N30	0.00																	
N31	0.61	0.00																
N24	0.88	0.97	0.00															
N32	0.75	0.89	0.94	0.00														
N33	0.75	1.00	0.97	0.72	0.00													
N34	0.75	1.00	0.97	0.71	1.00	0.00												
N35	0.55	1.00	0.97	0.80	1.00	1.00	0.00											
N13	0.89	0.95	0.95	0.94	0.94	0.94	0.94	0.00										
N15	0.89	0.98	0.97	0.95	0.98	0.98	0.98	0.73	0.00									
N16	0.89	1.00	0.97	0.95	1.00	1.00	1.00	0.61	0.92	0.00								
N17	0.89	1.00	0.97	0.94	1.00	1.00	1.00	0.75	0.92	1.00	0.00							
N14	0.89	0.98	0.97	0.95	0.98	0.98	0.98	0.85	0.95	0.96	0.94	0.00						
N25	0.90	0.95	0.94	0.95	0.94	0.94	0.94	0.93	0.94	0.94	0.94	0.95	0.00					
N29	0.91	0.94	0.93	0.94	0.93	0.93	0.92	0.93	0.93	0.93	0.93	0.94	0.86	0.00				
N27	0.89	1.00	0.97	0.95	1.00	1.00	1.00	0.93	0.98	1.00	1.00	0.98	0.84	0.84	0.00			
N26	0.90	0.97	0.97	0.95	0.97	0.97	0.97	0.95	0.97	0.97	0.97	0.97	0.89	0.88	0.93	0.00		
N8	0.91	0.97	0.98	0.95	0.97	0.97	0.97	0.95	0.98	0.97	0.97	0.98	0.95	0.94	0.97	0.97	0.00	

The significance level was 0.05.

## Discussion

The statistical parsimony analysis showed that of the 352 unique haplotypes, all but 15 belonged to the network that corresponded to one of the 24 putative species identified in Dinsdale et al. (2010) [27]. Of the 15 haplotypes, 3 belonged to new putative species identified using the rule set in Dinsdale et al. (2010) [27]; this was confirmed using statistical parsimony. The remaining 12 were considerably more divergent than the mean K2P divergences and occupied the upper end of the divergence range for the putative species from which they were split. Furthermore, of the six putative species, only two, AsiaII_7 and New World were shown to vary markedly from Dinsdale et al. (2010) [27]. This suggests that in most cases the K2P based approach devised by Dinsdale et al. (2010) and the TCS approach are in close agreement producing very similar assignment estimates which we know are well supported by available courtship and mating data [25], [28], [32], [33]. The approach also identified the need for more work, especially in the case of the New World to further resolve relationships. Our results further support the view [21] that when attempting to estimate species diversity where morphological data is unhelpful, network analysis is a good supporting tool that either confirms molecular phylogenetic analysis or shows where more work is needed to resolve the relationships That said, while network analytics provided good agreement with a phylogenetic approach, the issue of where to draw the line between species is complex. Multiple methods [46]–[55] have been applied to the questions of species delimitation, but there is no consensus as to which approach provides the best estimate or whether a best estimate is possible [56], [57]. Increasingly, there is a view that multiple genes are required to identify species level boundaries [58]–[61]. However, in the context of invasive species and biosecurity in general, species complexes such as that exemplified by *B. tabaci* which move around the world as a consequence of trade [39], [40] require decisions by regulators to be made quickly without the recourse to generating new data. In this study, the dataset that covers the widest available range of *B. tabaci* diversity is mtCOI and both a phylogenetic and network based analysis of this data provided similar outcomes that agree with available mating compatibility data. Future research could further consider the analysis of this dataset with a range of species distinctiveness measures to further explore the challenge of species delimitation and the applicability of the approaches used to date. The addition of genomic data will no doubt add further to our understanding of species level separation in this complex.

The results are based on a partial single maternally inherited gene (mtCOI) and what data there is supports a high level of congruence between phylogenetic relationships derived from mitochondrial and genomic DNA [25], [26]. Sole reliance on mtCOI as a means of considering the phylogeography of biological invasions is not unusual [62]–[64] although the addition of genomic DNA would add considerably to our understanding of the patterns of invasion. Furthermore, the *B. tabaci* complex shows very marked phylogeographic structure [26], [27] so in both cases the invasive MEAM1 and MED are genetically distinct and discrete when compared with the indigenous haplotypes in the geographies that have been invaded. The patterns of diversity and distribution of both globally invasive members of the *B. tabaci* cryptic species complex follow similar patterns. In both cases only a small number of haplotypes represent the majority of haplotypes that have spread beyond the home ranges; one haplotype in the case of MEAM1 and three in the case of MED account for >80% of the GenBank records in the invaded ranges. Furthermore, in both cases, the one haplotype for MEAM1 and two of the three for MED form hubs to which most invader haplotypes connect directly. The analysis of the haplotype data strongly suggests a role for Israel as the source of MEAM1 that has invaded most parts of the world whereas the pattern of invasion for MED suggests two sources. The first is the eastern Mediterranean where a haplotype found only in this part of MED's home range has been detected only the USA where it occurs primarily in Florida and to a lesser extent California. The second are haplotypes that occur in the western Mediterranean which have spread not only to the USA, but also to Asia and South America. It is also of some interest that in the case of MED 12 out of 86 haplotypes have spread beyond the assumed home range whereas for MEAM1 44 of the 80 haplotypes identified occurred outside the home range. Given that MEAM1 appears to have first begun to invade globally in the mid 1990s, around 10 years before MED began its global invasion, one might interpret this result in the context of the increasing body of knowledge that is accumulating in regards to global invasions which suggests that over time, invaded ranges accumulate an increasing proportion of the home range genetic diversity [62]–[68]. However, the above interpretations need to be considered with a degree of caution as the relative proportions of haplotypes that have spread beyond the home range may be an artifact of the varying levels of sampling effort across the different studies from which the sequences were sourced. In the case of MEAM1, the level of sampling across the home range would appear to be quite low as only 8.9% of sequences came from within the home range. This suggests that the invaded range has been subject to far more intensive sampling than the home range implying that the findings overestimate the proportion of diversity that has moved beyond the home ranges. In addition, the paucity of sampling within the home range of MEAM1 suggests the proposed origin of the global invasion needs to be considered with some caution. The situation for MED is somewhat different; the level of sampling across the home range is much higher as 59.1% of sequences came from within the home range. Furthermore, given that the numbers of sequences from the invaded and home ranges are similar, one might conclude that the levels of sampling effort across the two domains is equivalent and as only eight of the 86 MED haplotypes were detected outside the home range, the proportion of diversity that has so far invaded is quite low.

The structure of the MED network supports the existence of two distributions within the home range. The first is a Sub-Saharan range and the second a Mediterranean range. The significance of this is unclear, but one possible explanation is that MED evolved in Sub-Saharan Africa and then spread to the eastern Mediterranean and from there to the western Mediterranean. There is also the suggestion that MED has moved back to Sub-Saharan Africa from the Mediterranean region, possibly via Sudan and that this shift is coincident with the global spread of invasive MED that has been documented over the past 10 years [40] so it is possible that this latter return to Sub-Saharan Africa represents a very recent invasion.

The MEAM1 network is a little simpler to interpret and supports the view that it originated in the Middle East - Asia Minor region. There is also a case to suggest the involvement of Israel in the global spread of MEAM1, but this needs to be tempered in the light of the low level of sampling across the home range relative to the invaded range. However, all the haplotypes that have spread globally and have a connection back to the home range, do so via Israel. Of the countries in the home range Israel has by far the most developed ornamental plant export industry which accounts for a third of Israel's agricultural exports and ornamental plants are the primary means by which MEAM1 has been spread globally [35].

Networks are bidirectional in nature and so cannot directly show a hierarchical relationship. However, in some cases the pattern of the network can be used to propose a directional relationship. In our case the structure of the MEAM1 network supports the argument for a Middle East - Asia Minor origin [25]. Further, the two quite divergent haplotypes, Pak8 and Pak9 which at the 95% parsimony cutoff were shown not to be connected to the network, do connect at 93% and in both cases to the distal end of the home range branch at Sy (Syria). The structure of the branch supports the idea that MEAM1 may have originated in the region encompassed by Iran, Iraq, Pakistan and Syria and then spread into the Arabian Peninsula and from there to the rest of the world through Israel. However, the paucity of sampling across the home range requires that this interpretation be viewed with caution and requires more detailed and extensive sampling to verify.

Another feature of the MEAM1 network is the radiation of nodes about the Jap1 node. Here, when the branch containing haplotypes that originated only in the presumed home range are removed, of the 52 remaining nodes, 39 nodes were within one node of Jap1. Of these, 32 are known to occur only in locations outside the home range. There are several possible explanations. Firstly, this proliferation may be due to sequencing read errors. We cannot check for this as the trace files are largely unavailable. Secondly, they may simply reflect a difference in sampling effort with greater effort being apportioned to areas that have been invaded by MEAM1. This is quite plausible as the bulk of the literature pertaining to this pest come from countries where the pest has invaded.

The data and analyses presented here provide the first detailed consideration of the network relationship between different members of the *B. tabaci* cryptic species complex. In so doing it provides an insight into the patterns of global spread of two highly invasive members of the complex. Studies comparing mtCOI variation in home and invaded ranges have found that depending on the example, genetic diversity in the invaded range may be less, equivalent or exceed that observed in the home range [62]–[64], [67]. Where diversity is lower in the invaded range bottlenecks and drift are considered the underlying drivers [69], [70]. There is a general view that in the case of biological invasions, genetic diversity is reduced in the invaded ranges, but recent reviews [66], [69], [71] challenge this perception. In cases where levels of diversity are the same or equivalent, it is suggested that multiple introductions over time is a key contributor to invasion success and that the accumulation of diversity can act to facilitate the process of invasion [66], [69]. On the basis of mtCOI haplotype diversity, the results, bearing in mind the limitations associated with using data based on unknown sampling effort, suggest that diversity in the invaded range is lower in the case of MED than in the home range and most likely lower for MEAM1 given the low level of sampling in the home range. While, the addition of genomic DNA would add greater strength to the current observation, the observation that most of the diversity in the invaded ranges is represented by one haplotype in the case of MEAM1 and three for MED raises the question as to why this is the case. One possible explanation is that the invasion pathway has only had access to a very limited portion of the available genetic diversity for each invader. Alternatively, the invasive genotypes represented by the predominant mtCOI haplotypes may be associated with a set of traits that facilitate their ability to access the invasion pathway. Whatever the reason, if the accumulation of genetic diversity is a key factor in increasing invasion success [66], [67], then measures that restrict the recruitment of additional genetic diversity should be maintained even after establishment and spread has occurred, so as to avoid increasing the genetic diversity of two already damaging invasive pests.

## Materials and Methods

### Mitochondrial cytochrome oxidase one sequences from GenBank

GenBank was searched for all *B. tabaci* mtCOI sequences. This yielded a dataset of 2325 sequences. Sequences were then aligned using ClustalX 1.83 [41]. Sequences were aligned against AB204577 and at the 5′ end began with GAAAATTAGAGGTATTT and terminated at the 3′ end with TCCTTTCTTCTTCTGCGGT; a 657 bp fragment. Sequences with gaps, stop codons or shorter than 610 bp were removed yielding a final dataset of 1436 sequences of which 352 were unique. The consensus sequences from Dinsdale et al. (2010) [27] were used to assign sequences to the different putative species. Within each putative species the first 38 and the last nine bases are highly conserved. A representative of each unique haplotype together with the number of each sequence representing each haplotype recovered from GenBank is detailed in Table S1.

### Mitochondrial cytochrome oxidase 1 haplotype network analysis

We used the 198 unique *B. tabaci* mtCOI haplotypes from Dinsdale et al. (2010) [27] and added a further 154 unique haplotypes accessed from GenBank (Table S1); a total of 352 unique haplotypes. The sequences were analysed using statistical parsimony [23] with the program TCS v.1.21 [22]. The network connection limit was set at 95% following Hart and Sunday (2007) [20] and Chen et al. (2010) [21]. The resulting networks identify both the relationship between the different haplotypes as well as the significant number of substitutions [23] connecting haplotypes. Readers wishing to see the relationship between the species names used here and biotype terminology are directed to the review De Barro et al. (2011) [25].

### Haplotype diversity and distribution for globally invasive *B. tabaci*

There are two globally invasive members of the *B. tabaci* complex MEAM1 and MED. In GenBank there were 660 MEAM1 sequences and 374 MED sequences and for each sequence the collection country location was then recorded. Duplicate haplotypes were identified using TCS [22]. A key point to bear in mind is that the sequences from GenBank are the results of numerous studies where the sampling effort used will be variable and the results should be viewed accordingly. The nucleotide composition and K2P genetic distance [42] between networks were calculated in MEGA 4.0 [43].

To identify limits to gene flow among these putative cryptic species, pairwise Φst values were calculated [44] and an analysis of molecular variance (AMOVA) undertaken using Arlequin v.3.1 [45].

## Supporting Information

Table S1 The 352 unique mitochondrial cytochrome oxidase 1 haplotypes used in the network analysis. The GenBank accession number for each haplotype is provided along with the country of origin. The haplotype identifier refers to haplotypes belonging to MEAM1 and MED where there were at least two identical sequences in GenBank; the haplotype identifiers are used in Figs 1, 2, 3. The network code indicates the abbreviation used in the networks presented in Figs 1, 2, 3. The number of sequences representing each haplotype is also provided. The haplotype identifier indicates a haplotype from either the Mediterranean or Middle East - Asia Minor 1 putative species which was represented by at least two accessions in GenBank. The network code is the identifier used for each accession in the network analysis.
(DOCX)
