# Supplementary material for: Genetic Networking of the Bemisia tabaci Cryptic Species Complex Reveals Pattern of Biological Invasions
Source: PLoS One. 2011 Oct 3;6(10):e25579. doi: 10.1371/journal.pone.0025579 (PMC3184991; doi:10.1371/journal.pone.0025579)
Supplement: Table S1 — The 352 unique mitochondrial cytochrome oxidase 1 haplotypes used in the network analysis. The GenBank accession number for each haplotype is provided along with the country of origin. The haplotype identifier refers to haplotypes belonging to MEAM1 and MED where there were at least two identical sequences in GenBank; the haplotype identifiers are used in Figs 1, 2, 3. The network code indicates the abbreviation used in the networks presented in Figs 1, 2, 3. The number of sequences representing each haplotype is also provided. The haplotype identifier indicates a haplotype from either the Mediterranean or Middle East - Asia Minor 1 putative species which was represented by at least two accessions in GenBank. The network code is the identifier used for each accession in the network analysis. (DOCX) [file pone.0025579.s001.docx]

**Table S1**. The 352 unique mitochondrial cytochrome oxidase 1 haplotypes used in the network analysis. The GenBank accession number for each haplotype is provided along with the country of origin. The haplotype identifier refers to haplotypes belonging to MEAM1 and MED where there were at least two identical sequences in GenBank; the haplotype identifiers are used in Figs 1-3. The network code indicates the abbreviation used in the networks presented in Figs 1-3. The number of sequences representing each haplotype is also provided. The haplotype identifier indicates a haplotype from either the Mediterranean or Middle East - Asia Minor 1 putative species which was represented by at least two accessions in GenBank. The network code is the identifier used for each accession in the network analysis.

| **Dinsdale species based on 3.5% sequence divergence** | **Accession number** | **Country of origin** | **Haplotype identifier** | **Network code** | **Number of sequences in GenBank** |
| --- | --- | --- | --- | --- | --- |
| AsiaI | AJ748359 | India |  | A1Ind1 | 1 |
| AsiaI | AJ748360 | India |  | A1Ind2 | 1 |
| AsiaI | AJ748365 | India |  | A1Ind3 | 1 |
| AsiaI | AJ510066 | Pakistan |  | A1Pak2 | 1 |
| AsiaI | AJ748369 | India |  | A1Ind4 | 1 |
| AsiaI | AJ748370 | India |  | A1Ind5 | 1 |
| AsiaI | AJ748371 | India |  | A1Ind6 | 1 |
| AsiaI | AJ748361 | India |  | A1Ind7 | 17 |
| AsiaI | AB248260 | Indonesia |  | A1Indo1 | 1 |
| AsiaI | AY686095 | Singapore |  | A1Sing | 1 |
| AsiaI | AB248262 | Indonesia |  | A1Indo2 | 1 |
| AsiaI | AY686093 | Malaysia |  | A1Mal1 | 1 |
| AsiaI | AJ510061 | Pakistan |  | A1Pak1 | 1 |
| AsiaI | AJ510069 | Pakistan |  | A1Pak3 | 1 |
| AsiaI | AJ510078 | Pakistan |  | A1Pak4 | 1 |
| AsiaI | AF164671 | Thailand |  | A1Thai1 | 2 |
| AsiaI | DQ986959 | China |  | A1Ch1 | 1 |
| AsiaI | EU192044 | China |  | A1Ch2 | 1 |
| AsiaI | EF528552 | Thailand |  | A1Thai2 | 1 |
| AsiaII_1 | AJ867557 | China |  | A2Ch1 | 11 |
| AsiaII_1 | DQ309077 | China |  | A2Ch2 | 2 |
| AsiaII_1 | EU192047 | China |  | A2Ch3 | 1 |
| AsiaII_1 | EU192065 | China |  | A2Ch4 | 1 |
| AsiaII_1 | AJ510058 | Pakistan |  | A2Pak1 | 3 |
| AsiaII_1 | AJ510057 | Pakistan |  | A2Pak2 | 1 |
| AsiaII_1 | AJ510065 | Pakistan |  | A2Pak3 | 1 |
| AsiaII_1 | AY686094 | Pakistan |  | A2Pak4 | 1 |
| AsiaII_1 | FJ710473 | Pakistan |  | A2Pak5 | 1 |
| AsiaII_1 | GU585375 | Pakistan |  | A2Pak6 | 1 |
| AsiaII_1 | GU585369 | Pakistan |  | A2Pak7 | 1 |
| AsiaII_1 | GU585370 | Pakistan |  | A2Pak8 | 1 |
| AsiaII_1 | GU585373 | Pakistan |  | A2Pak9 | 1 |
| AsiaII_1 | FJ802389 | Syria |  | A2Sy1 | 1 |
| AsiaII_1 | FJ802393 | Syria |  | A2Sy2 | 1 |
| AsiaII_2 | AY686088 | China |  | A2Ch5 | 1 |
| AsiaII_3 | DQ309075 | China |  | A2Ch6 | 1 |
| AsiaII_3 | EU192045 | China |  | A2Ch7 | 1 |
| AsiaII_3 | AJ783706 | China |  | A2Ch8 | 19 |
| AsiaII_3 | AJ867556 | China |  | A2Ch9 | 1 |
| AsiaII_4 | AY686083 | China |  | A2Ch10 | 1 |
| AsiaII_5 | AJ748376 | India |  | A2Ind1 | 2 |
| AsiaII_5 | AF418666 | India |  | A2Ind2 | 3 |
| AsiaII_6 | AJ784261 | China |  | A2Ch11 | 2 |
| AsiaII_6 | EU192052 | China |  | A2Ch12 | 1 |
| AsiaII_6 | EU192054 | China |  | A2Ch13 | 10 |
| AsiaII_7 | AY686064 | China |  | Chi1 | 1 |
| AsiaII_7 | AY686075 | Taiwan |  | Tai1 | 1 |
| AsiaII_7 | AJ748375 | India |  | Ind1 | 1 |
| AsiaII_7 | AJ748372 | India |  | Ind2 | 1 |
| AsiaII_7 | DQ116660 | India |  | Ind3 | 1 |
| AsiaII_7 | DQ174523 | China |  | Ch2 | 1 |
| AsiaII_7 | AJ748378 | India |  | Ind4 | 6 |
| AsiaII_7 | DQ116661 | India |  | Ind5 | 2 |
| AsiaII_7 | DQ116650 | India |  | Ind6 | 1 |
| AsiaII_7 | DQ116662 | India |  | Ind7 | 1 |
| AsiaII_7 | DQ174521 | Taiwan |  | Tai2 | 1 |
| AsiaII_7 | AM408899 | India |  | Ind8 | 1 |
| AsiaII_8 | AJ748358 | India |  | A2Ind7 | 1 |
| AsiaII_8 | AJ748374 | India |  | A2Ind8 | 1 |
| AsiaII_8 | AJ748357 | India |  | A2Ind9 | 3 |
| AsiaII_8 | AJ748362 | India |  | A2Ind10 | 4 |
| Australia | GU086328 | Australia |  | Aus1 | 2 |
| Australia_Indonesia | HQ457045 | Indonesia |  | Aus2 | 2 |
| Australia_Indonesia | GU086325 | Indonesia |  | Aus3 | 1 |
| Australia_Indonesia | GU086326 | Indonesia |  | Aus4 | 1 |
| Australia_Indonesia | GU086327 | Indonesia |  | Aus5 | 1 |
| China1 | AY686085 | China |  | Ch1 | 1 |
| China1 | AY686089 | China |  | Ch2 | 1 |
| China1 | AY686091 | China |  | Ch3 | 1 |
| China1 | GQ139495 | China |  | Ch5 | 1 |
| China1 | GQ303180 | China |  | Ch6 | 9 |
| China2 | AY686072 | China |  | Ch4 | 1 |
| ex China | EU192051 | China |  | Ch7 | 1 |
| Indian Ocean | AJ550171 | Madagascar |  | IOMad | 4 |
| Indian Ocean | AJ550178 | Reunion |  | IOR1 | 1 |
| Indian Ocean | AJ550179 | Reunion |  | IOR2 | 1 |
| Indian Ocean | AJ550180 | Reunion |  | IOR3 | 1 |
| Indian Ocean | AY903522 | Uganda |  | IOU1 | 1 |
| Indian Ocean | AY903523 | Uganda |  | IOU2 | 1 |
| Indian Ocean | AY903525 | Uganda |  | IOU3 | 2 |
| Indian Ocean | AY903536 | Uganda |  | IOU4 | 1 |
| Indian Ocean | AY903537 | Uganda |  | IOU5 | 2 |
| Indian Ocean | AY903538 | Uganda |  | IOU6 | 1 |
| Indian Ocean | AY903539 | Uganda |  | IOU7 | 1 |
| Indian Ocean | AY903527 | Uganda |  | IOU8 | 1 |
| Mediterranean | GU086329 | China | Hap1 | MCh1 | 101 |
| Mediterranean | GU086333 | China | Hap2 | MCh2 | 35 |
| Mediterranean | AY827617 | Turkey | Hap3 | MTur1 | 43 |
| Mediterranean | GU086330 | Czech Republic | Hap4 | MCB | 14 |
| Mediterranean | FJ766432 | Burkina Faso | Hap5 | MBF1 | 3 |
| Mediterranean | AY057136 | Ivory Coast, SL | Hap6 | MIC | 13 |
| Mediterranean | GU086332 | China | Hap7 | MCh3 | 6 |
| Mediterranean | DQ133378 | Sudan1 | Hap8 | MSu1 | 19 |
| Mediterranean | FJ766391 | Burkina Faso | Hap9 | MBF2 | 11 |
| Mediterranean | FJ766400 | Burkina Faso | Hap10 | MBF3 | 6 |
| Mediterranean | FJ766387 | Burkina Faso | Hap11 | MBF4 | 8 |
| Mediterranean | FJ766405 | Burkina Faso | Hap12 | MBF5 | 2 |
| Mediterranean | AF342773 | Morocco | Hap13 | MMor1 | 3 |
| Mediterranean | FJ025797 | Egypt | Hap14 | MEg1 | 4 |
| Mediterranean | AY827606 | Nigeria, SL | Hap15 | MNig | 13 |
| Mediterranean | DQ365857 | Crete | Hap16 | MCre1 | 12 |
| Mediterranean | AF342775 | Spain | Hap17 | MSp1 | 3 |
| Mediterranean | DQ365856 | Crete | Hap18 | MCre2 | 4 |
| Mediterranean | AM691059 | France | Hap19 | MFra1 | 3 |
| Mediterranean | EU760721 | Cameroon | Hap20 | MCam1 | 2 |
| Mediterranean | AM691062 | France | Hap21 | MFra2 | 2 |
| Mediterranean | AY903540 | Uganda | Hap22 | MUg1 | 2 |
| Mediterranean | AB297897 | Syria | Hap23 | MSy1 | 2 |
| Mediterranean | AY903565 | Uganda |  | MUg7 | 1 |
| Mediterranean | AM176574 | Algeria |  | MAlg1 | 1 |
| Mediterranean | AM176575 | Algeria |  | MAlg2 | 1 |
| Mediterranean | FJ766381 | Burkina Faso |  | MBF6 | 1 |
| Mediterranean | FJ766384 | Burkina Faso |  | MBF7 | 1 |
| Mediterranean | FJ766385 | Burkina Faso |  | MBF8 | 1 |
| Mediterranean | FJ766408 | Burkina Faso |  | MBF9 | 1 |
| Mediterranean | FJ766429 | Burkina Faso |  | MBF10 | 1 |
| Mediterranean | FJ766383 | Ivory Coast |  | MIC1 | 1 |
| Mediterranean | GU086334 | Croatia |  | MCro1 | 1 |
| Mediterranean | GU086335 | Croatia |  | MCro2 | 1 |
| Mediterranean | GU086336 | Croatia |  | MCro3 | 1 |
| Mediterranean | GU086337 | Croatia |  | MCro4 | 1 |
| Mediterranean | GU086338 | Croatia |  | MCro5 | 1 |
| Mediterranean | EF694112 | China |  | MCh4 | 1 |
| Mediterranean | EU192061 | China |  | MCh5 | 1 |
| Mediterranean | AM180063 | France |  | MFra3 | 1 |
| Mediterranean | AM691055 | France |  | MFra4 | 1 |
| Mediterranean | AM691063 | France |  | MFra5 | 1 |
| Mediterranean | AM691064 | France |  | MFra6 | 1 |
| Mediterranean | GU086331 | Egypt |  | MFra7 | 1 |
| Mediterranean | AJ517769 | Morocco |  | MMor2 | 1 |
| Mediterranean | AM176571 | Morocco |  | MMor3 | 1 |
| Mediterranean | AM176573 | Morocco |  | MMor4 | 1 |
| Mediterranean | AY057138 | Morocco |  | MMor5 | 1 |
| Mediterranean | AF342769 | Spain |  | MSp2 | 1 |
| Mediterranean | DQ174539 | Spain |  | MSp3 | 1 |
| Mediterranean | DQ174540 | Spain |  | MSp4 | 1 |
| Mediterranean | AY827579 | Ghana, SL |  | MGh1 | 1 |
| Mediterranean | AY827580 | Ghana, SL |  | MGh2 | 1 |
| Mediterranean | AY827582 | Ghana, SL |  | MGh3 | 1 |
| Mediterranean | AY827588 | Ghana, SL |  | MGh4 | 1 |
| Mediterranean | AY827589 | Ghana, SL |  | MGh5 | 1 |
| Mediterranean | AY827590 | Ghana, SL |  | MGh6 | 1 |
| Mediterranean | AY827612 | Sudan |  | MSu2 | 1 |
| Mediterranean | AY827613 | Sudan |  | MSu3 | 1 |
| Mediterranean | AY827614 | Sudan |  | MSu4 | 1 |
| Mediterranean | AY827615 | Sudan |  | MSu5 | 1 |
| Mediterranean | AB297898 | Syria |  | MSy2 | 1 |
| Mediterranean | GU086339 | Taiwan |  | MTai | 1 |
| Mediterranean | AY827619 | Turkey |  | MTur2 | 1 |
| Mediterranean | AY903578 | Uganda |  | MUg2 | 1 |
| Mediterranean | AF344258 | Cameroon |  | MCam2 | 1 |
| Mediterranean | AF344285 | Zimbabwe |  | MZim1 | 1 |
| Mediterranean | AF344286 | Zimbabwe |  | MZm2 | 1 |
| Mediterranean | AM040609 | Ghana |  | MGh7 | 1 |
| Mediterranean | AM176572 | Morocco |  | MMor6 | 1 |
| Mediterranean | AY827581 | Ghana |  | MGh8 | 1 |
| Mediterranean | AY903529 | Uganda |  | MUg3 | 1 |
| Mediterranean | AY903531 | Uganda |  | MUg4 | 1 |
| Mediterranean | AY903534 | Uganda |  | MUg5 | 1 |
| Mediterranean | AY903551 | Uganda |  | MKab | 1 |
| Mediterranean | AY903556 | Uganda |  | MMuk | 1 |
| Mediterranean | AY903564 | Uganda |  | MUg6 | 1 |
| Mediterranean | DQ302946 | Spain |  | MSp5 | 1 |
| Mediterranean | EF667476 | China |  | MCh6 | 1 |
| Mediterranean | EF694108 | Egypt |  | MEg2 | 1 |
| Mediterranean | EF694111 | China |  | MCh7 | 1 |
| Mediterranean | EU192049 | China |  | MCh8 | 1 |
| Mediterranean | EU760741 | Cameroon |  | MCam3 | 1 |
| Mediterranean | FJ766431 | Burkino Faso |  | MBF11 | 1 |
| Mediterranean | GQ139500 | China |  | MCh9 | 1 |
| Mediterranean | GU168793 | China |  | MCh10 | 1 |
| Italy | AY827596 | Sicily |  | Italy1 | 1 |
| Italy | AY827598 | Sicily |  | Italy2 | 1 |
| Italy | AY827599 | Sicily |  | Italy3 | 1 |
| Italy | AY827600 | Sicily |  | Italy4 | 1 |
| Italy | AY827601 | Sicily |  | Italy5 | 1 |
| Italy | AY827602 | Sicily |  | Italy6 | 1 |
| Italy | AY827603 | Sicily |  | Italy7 | 1 |
| Middle East - Asia Minor 1 | AB204577 | Japan | Hap1 | Jap1 | 527 |
| Middle East - Asia Minor 1 | AB473559 | Syria | Hap2 | Sy | 10 |
| Middle East - Asia Minor 1 | AJ510071 | Pakistan | Hap3 | Pak1 | 4 |
| Middle East - Asia Minor 1 | AY686063 | China | Hap4 | Ch1 | 24 |
| Middle East - Asia Minor 1 | AF340215 | Argentina | Hap5 | Arg | 11 |
| Middle East - Asia Minor 1 | EF398087 | China | Hap6 | Ch2 | 4 |
| Middle East - Asia Minor 1 | AF418671 | Israel | Hap7 | Is1 | 2 |
| Middle East - Asia Minor 1 | DQ174538 | South Korea | Hap8 | SK | 3 |
| Middle East - Asia Minor 1 | GU977269 | Pakistan | Hap9 | Pak2 | 2 |
| Middle East - Asia Minor 1 | AJ510076 | Pakistan | Hap10 | Pak3 | 2 |
| Middle East - Asia Minor 1 | DQ989539 | Taiwan | Hap11 | Tai1 | 2 |
| Middle East - Asia Minor 1 | AB204578 | Japan |  | Jap2 | 1 |
| Middle East - Asia Minor 1 | AB204580 | Japan |  | Jap3 | 1 |
| Middle East - Asia Minor 1 | AB204581 | Japan |  | Jap4 | 1 |
| Middle East - Asia Minor 1 | AJ510075 | Pakistan |  | Pak4 | 1 |
| Middle East - Asia Minor 1 | AJ510079 | Pakistan |  | Pak5 | 1 |
| Middle East - Asia Minor 1 | AJ510081 | Pakistan |  | Pak6 | 1 |
| Middle East - Asia Minor 1 | AJ517768 | Morocco |  | Mor1 | 1 |
| Middle East - Asia Minor 1 | AJ550173 | Mayotte |  | May | 1 |
| Middle East - Asia Minor 1 | AJ550174 | Reunion |  | Reu1 | 1 |
| Middle East - Asia Minor 1 | AJ748368 | India |  | Ind1 | 1 |
| Middle East - Asia Minor 1 | AJ877260 | Reunion |  | Reu2 | 1 |
| Middle East - Asia Minor 1 | AM040594 | India |  | Ind2 | 1 |
| Middle East - Asia Minor 1 | AM176570 | Morocco |  | Mor2 | 1 |
| Middle East - Asia Minor 1 | AM180064 | Guadeloupe |  | SF | 1 |
| Middle East - Asia Minor 1 | AM408896 | India |  | Ind3 | 1 |
| Middle East - Asia Minor 1 | HM070414 | Emerald |  | Aus1 | 1 |
| Middle East - Asia Minor 1 | AY057123 | USA |  | USA1 | 1 |
| Middle East - Asia Minor 1 | AY686062 | China |  | Ch3 | 1 |
| Middle East - Asia Minor 1 | AY686073 | China |  | Ch4 | 1 |
| Middle East - Asia Minor 1 | AY686078 | China |  | Ch5 | 1 |
| Middle East - Asia Minor 1 | AY747688 | Israel |  | Is2 | 1 |
| Middle East - Asia Minor 1 | HM070412 | Israel |  | Is3 | 1 |
| Middle East - Asia Minor 1 | AY766373 | Israel |  | Is4 | 1 |
| Middle East - Asia Minor 1 | DQ133382 | United Arab Emirates |  | UAE | 1 |
| Middle East - Asia Minor 1 | DQ174530 | Taiwan |  | Tai2 | 1 |
| Middle East - Asia Minor 1 | DQ174534 | Taiwan |  | Tai3 | 1 |
| Middle East - Asia Minor 1 | DQ174535 | Australia |  | Aus2 | 1 |
| Middle East - Asia Minor 1 | DQ174536 | Israel |  | Is5 | 1 |
| Middle East - Asia Minor 1 | DQ174537 | Japan |  | Jap5 | 1 |
| Middle East - Asia Minor 1 | DQ989525 | China |  | Ch6 | 1 |
| Middle East - Asia Minor 1 | EF398086 | China |  | Ch7 | 1 |
| Middle East - Asia Minor 1 | EF398088 | China |  | Ch8 | 1 |
| Middle East - Asia Minor 1 | EF398090 | China |  | Ch9 | 1 |
| Middle East - Asia Minor 1 | EF398091 | China |  | Ch10 | 1 |
| Middle East - Asia Minor 1 | EF398113 | China |  | Ch11 | 1 |
| Middle East - Asia Minor 1 | EF398127 | Israel |  | Is6 | 1 |
| Middle East - Asia Minor 1 | EU192068 | China |  | Ch12 | 1 |
| Middle East - Asia Minor 1 | EU263624 | China |  | Ch13 | 1 |
| Middle East - Asia Minor 1 | EU263625 | China |  | Ch14 | 1 |
| Middle East - Asia Minor 1 | EU547770 | Iran |  | Ir1 | 1 |
| Middle East - Asia Minor 1 | GQ332577 | China |  | Ch15 | 1 |
| Middle East - Asia Minor 1 | GU086341 | Italy |  | It | 1 |
| Middle East - Asia Minor 1 | GU086342 | Taiwan |  | Tai4 | 1 |
| Middle East - Asia Minor 1 | GU086343 | Yemen |  | Yem1 | 1 |
| Middle East - Asia Minor 1 | GU086344 | Saudi Arabia |  | SA1 | 1 |
| Middle East - Asia Minor 1 | GU086345 | Saudi Arabia |  | SA2 | 1 |
| Middle East - Asia Minor 1 | GU086346 | Kuwait |  | Ku1 | 1 |
| Middle East - Asia Minor 1 | GU086347 | Jordan |  | Jor | 1 |
| Middle East - Asia Minor 1 | GU086348 | Spain |  | Sp | 1 |
| Middle East - Asia Minor 1 | GU086349 | Dominican Republic |  | DRP | 1 |
| Middle East - Asia Minor 1 | GU086350 | Iran |  | Ir2 | 1 |
| Middle East - Asia Minor 1 | GU086351 | Iran |  | Ir3 | 1 |
| Middle East - Asia Minor 1 | GU086352 | Iran |  | Ir4 | 1 |
| Middle East - Asia Minor 1 | GU086353 | Iran |  | Ir5 | 1 |
| Middle East - Asia Minor 1 | GU086354 | Kuwait |  | Ku2 | 1 |
| Middle East - Asia Minor 1 | GU086355 | Kuwait |  | Ku3 | 1 |
| Middle East - Asia Minor 1 | GU086356 | Kuwait |  | Ku4 | 1 |
| Middle East - Asia Minor 1 | GU086357 | Saudi Arabia |  | SA3 | 1 |
| Middle East - Asia Minor 1 | GU086358 | Saudi Arabia |  | SA4 | 1 |
| Middle East - Asia Minor 1 | GU086359 | Yemen |  | Yem2 | 1 |
| Middle East - Asia Minor 1 | GU086360 | Yemen |  | Yem3 | 1 |
| Middle East - Asia Minor 1 | HM070410 | Indonesia |  | Indo | 1 |
| Middle East - Asia Minor 1 | HM070413 | Iraq |  | Iraq | 1 |
| Middle East - Asia Minor 1 | HM070411 | USA |  | USA2 | 1 |
| Middle East - Asia Minor 1 | GU977249 | Egypt |  | Eg | 1 |
| Middle East - Asia Minor 1 | GU977273 | Pakistan |  | Pak7 | 1 |
| Middle East - Asia Minor 1 | EF398083 | China |  | Ch16 | 1 |
| Middle East - Asia Minor 1 | GU977268 | Pakistan |  | Pak8 | 1 |
| Middle East - Asia Minor 1 | GU977267 | Pakistan |  | Pak9 | 1 |
| Middle East - Asia Minor 2 | AJ550177 | Reunion |  | R3 | 1 |
| New World 1 | DQ130060 | Panama |  | NEWP1 | 1 |
| New World 1 | EU760727 | Sudan |  | NEWSD | 2 |
| New World 1 | AJ550167 | Colombia |  | NWC1 | 1 |
| New World 1 | AJ550168 | Colombia |  | NEWC2 | 1 |
| New World 1 | EU427728 | Colombia |  | NEWC3 | 1 |
| New World 1 | AY057134 | Puerto Rico |  | NEWPR | 1 |
| New World 1 | AY057129 | Guatemala |  | NEWG | 1 |
| New World 1 | AY057128 | El Salvador |  | NEWES | 1 |
| New World 1 | DQ130053 | Belize |  | NEWB | 2 |
| New World 1 | AY057126 | Mexico |  | NEWM1 | 1 |
| New World 1 | DQ130058 | Mexico |  | NEWM2 | 1 |
| New World 1 | DQ130059 | Mexico |  | NEWM3 | 1 |
| New World 1 | EU427729 | Mexico |  | NEWM4 | 9 |
| New World 1 | DQ130061 | Panama |  | NEWP2 | 1 |
| New World 1 | AY521259 | USA |  | NEWUS | 2 |
| New World 1 | AY057133 | Honduras |  | NEWH1 | 1 |
| New World 2 | AF340212 | Argentina |  | NEWA1 | 1 |
| New World 2 | AF340213 | Argentina |  | NEWA2 | 2 |
| Sub-Saharan Africa 1 | AY827591 | Ghana |  | G1 | 1 |
| Sub-Saharan Africa 1 | AF418668 | Ghana |  | G2 | 4 |
| Sub-Saharan Africa 1 | AY057149 | Uganda |  | U1 | 3 |
| Sub-Saharan Africa 1 | AY057151 | Uganda |  | U2 | 2 |
| Sub-Saharan Africa 1 | AY057169 | Uganda |  | U3 | 1 |
| Sub-Saharan Africa 1 | AY057180 | Uganda |  | U4 | 43 |
| Sub-Saharan Africa 1 | AY057162 | Malawi |  | MA | 1 |
| Sub-Saharan Africa 1 | AF344278 | Mozambique |  | MO | 1 |
| Sub-Saharan Africa 1 | AF344264 | South Africa |  | SA1 | 1 |
| Sub-Saharan Africa 1 | AF344267 | South Africa |  | SA2 | 1 |
| Sub-Saharan Africa 1 | AF344276 | Swaziland |  | SW | 1 |
| Sub-Saharan Africa 1 | AF418667 | Tanzania |  | T | 1 |
| Sub-Saharan Africa 1 | AY057168 | Uganda |  | U5 | 1 |
| Sub-Saharan Africa 1 | AY057178 | Uganda |  | U6 | 1 |
| Sub-Saharan Africa 1 | AY057179 | Uganda |  | U7 | 1 |
| Sub-Saharan Africa 1 | AY057181 | Uganda |  | U8 | 1 |
| Sub-Saharan Africa 1 | AY057182 | Uganda |  | U9 | 1 |
| Sub-Saharan Africa 1 | AY057183 | Uganda |  | U10 | 1 |
| Sub-Saharan Africa 1 | AY057185 | Uganda |  | U11 | 1 |
| Sub-Saharan Africa 1 | AY057210 | Uganda |  | U12 | 1 |
| Sub-Saharan Africa 1 | AY903461 | Uganda |  | U19 | 1 |
| Sub-Saharan Africa 1 | AY903512 | Uganda |  | U21 | 3 |
| Sub-Saharan Africa 1 | AY903493 | Uganda |  | U22 | 1 |
| Sub-Saharan Africa 1 | AY903494 | Uganda |  | U23 | 1 |
| Sub-Saharan Africa 1 | AY903502 | Uganda |  | U24 | 1 |
| Sub-Saharan Africa 1 | AY903507 | Uganda |  | U25 | 1 |
| Sub-Saharan Africa 1 | AY903510 | Uganda |  | U26 | 1 |
| Sub-Saharan Africa 1 | AY903511 | Uganda |  | U27 | 1 |
| Sub-Saharan Africa 1 | AY903462 | Uganda |  | U28 | 41 |
| Sub-Saharan Africa 1 | AY903464 | Uganda |  | U29 | 1 |
| Sub-Saharan Africa 1 | AY903483 | Uganda |  | B1 | 1 |
| Sub-Saharan Africa 1 | AY903487 | Uganda |  | B2 | 1 |
| Sub-Saharan Africa 1 | AY903470 | Uganda |  | U30 | 1 |
| Sub-Saharan Africa 1 | AY903475 | Uganda |  | U31 | 1 |
| Sub-Saharan Africa 1 | AY903466 | Uganda |  | U32 | 1 |
| Sub-Saharan Africa 1 | AY903469 | Uganda |  | U33 | 1 |
| Sub-Saharan Africa 1 | AY903479 | Uganda |  | U34 | 1 |
| Sub-Saharan Africa 1 | AY903490 | Uganda |  | U36 | 1 |
| Sub-Saharan Africa 1 | AY903492 | Uganda |  | U37 | 1 |
| Sub-Saharan Africa 1 | AY903519 | Uganda |  | U38 | 2 |
| Sub-Saharan Africa 1 | AY563641 | Uganda |  | U39 | 1 |
| Sub-Saharan Africa 1 | AY563648 | Uganda |  | U41 | 2 |
| Sub-Saharan Africa 1 | AY563652 | Uganda |  | U42 | 1 |
| Sub-Saharan Africa 1 | AY563657 | Uganda |  | U43 | 1 |
| Sub-Saharan Africa 1 | AY563666 | Uganda |  | U45 | 1 |
| Sub-Saharan Africa 1 | AY563674 | Uganda |  | U47 | 1 |
| Sub-Saharan Africa 1 | AY563679 | Uganda |  | U48 | 1 |
| Sub-Saharan Africa 1 | AY563702 | Uganda |  | U50 | 1 |
| Sub-Saharan Africa 2 | GU086361 | Spain |  | P1 | 4 |
| Sub-Saharan Africa 2 | AY827611 | Spain |  | P2 | 1 |
| Sub-Saharan Africa 2 | AY827605 | Mali |  | ML2 | 1 |
| Sub-Saharan Africa 2 | AY057141 | Uganda |  | U14 | 3 |
| Sub-Saharan Africa 2 | AY057173 | Uganda |  | U17 | 3 |
| Sub-Saharan Africa 2 | AY057194 | Uganda |  | U18 | 2 |
| Sub-Saharan Africa 2 | AY563646 | Uganda |  | U40 | 13 |
| Sub-Saharan Africa 2 | AY057143 | Uganda |  | U15 | 2 |
| Sub-Saharan Africa 2 | AY057146 | Uganda |  | U16 | 2 |
| Sub-Saharan Africa 2 | AY563664 | Uganda |  | U44 | 1 |
| Sub-Saharan Africa 2 | AY563667 | Uganda |  | U46 | 1 |
| Sub-Saharan Africa 2 | AY827604 | Mali |  | ML1 | 1 |
| Sub-Saharan Africa 2 | AY827607 | Nigeria |  | MN | 1 |
| Sub-Saharan Africa 3 | AF344257 | Cameroon |  | C1 | 1 |
| Sub-Saharan Africa 4 | AF344245 | Cameroon |  | C2 | 1 |
| Sub-Saharan Africa 4 | AF344247 | Cameroon |  | C3 | 1 |
| Sub-Saharan Africa 4 | AF344249 | Cameroon |  | C4 | 1 |
| Sub-Saharan Africa 4 | AF344251 | Cameroon |  | C5 | 1 |
| Sub-Saharan Africa 4 | AF344252 | Cameroon |  | C6 | 1 |
| Sub-Saharan Africa 4 | AF344254 | Cameroon |  | C7 | 1 |
| Sub-Saharan Africa 4 | AF344255 | Cameroon |  | C8 | 1 |
| Sub-Saharan Africa 4 | AF344246 | Cameroon |  | C9 | 1 |
| Uganda | AF418665 | Uganda |  | UG1 | 4 |
| Uganda | AY903576 | Uganda |  | UG2 | 1 |
| Uganda | AY903577 | Uganda |  | UG3 | 1 |
| Uganda | AY903579 | Uganda |  | UG4 | 1 |
| Uganda | AY903553 | Uganda |  | UG5 | 1 |
